# Mn doped Prussian blue nanoparticles for T_1_/T_2_ MR imaging, PA imaging and Fenton reaction enhanced mild temperature photothermal therapy of tumor

**DOI:** 10.1186/s12951-021-01235-2

**Published:** 2022-01-04

**Authors:** Quan Tao, Genghan He, Sheng Ye, Di Zhang, Zhide Zhang, Li Qi, Ruiyuan Liu

**Affiliations:** Guangdong Provincial Key Laboratory of Medical Image Processing, School of Biomedical Engineering, Southern Medical University, Guangzhou, 510515 Guangdong China

**Keywords:** MR imaging, Photoacoustic imaging, Mild temperature photothermal therapy, Chemodynamic therapy, Fenton reaction

## Abstract

**Background:**

Combining the multimodal imaging and synergistic treatment in one platform can enhance the therapeutic efficacy and diagnosis accuracy.

**Results:**

In this contribution, innovative Mn-doped Prussian blue nanoparticles (MnPB NPs) were prepared via microemulsion method. MnPB NPs demonstrated excellent T_1_ and T_2_ weighted magnetic resonance imaging (MRI) enhancement in vitro and in vivo. The robust absorbance in the near infrared range of MnPB NPs provides high antitumor efficacy for photothermal therapy (PTT) and photoacoustics imaging property. Moreover, with the doping of Mn, MnPB NPs exhibited excellent Fenton reaction activity for chemodynamic therapy (CDT). The favorable trimodal imaging and Fenton reaction enhanced mild temperature photothermal therapy in vitro and in vivo were further confirmed that MnPB NPs have significant positive effectiveness for integration of diagnosis and treatment tumor.

**Conclusions:**

Overall, this Mn doped Prussian blue nanoplatform with multimodal imaging and chemodynamic/mild temperature photothermal co-therapy provides a reliable tool for tumor treatment.

**Graphical Abstract:**

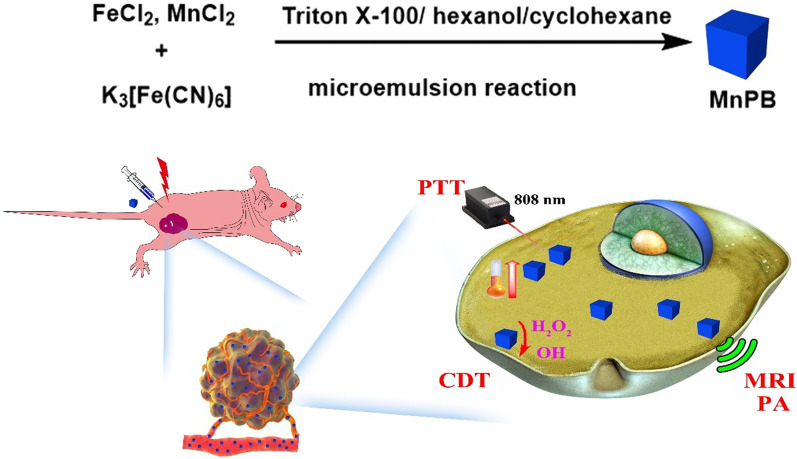

**Supplementary Information:**

The online version contains supplementary material available at 10.1186/s12951-021-01235-2.

## Introduction

Photothermal therapy (PTT) is a therapeutic intervention treatment model for tumors, which employs near-infrared absorption materials to produce heat for tumor ablation [1–9]. The generated hyperthermia can effectively and rapidly ablate tumor without systemic toxicity and minimize the invasion. Nevertheless, the extreme high temperature from PTT (> 50 ℃) may bring collateral damage to surrounding normal tissues by heat diffusion. Moreover, photothermal agents with high concentration in tumor or high NIR laser intensity is required to generate hyperthermia, which may increase health risks due to drug overdose or photodamage. Recently, mild temperature hyperthermia (< 48 ℃) has attracted great attention due to the excellent tolerance and safety for surrounding tissues [10–12]. However, the weak treatment effectiveness of mild temperature hyperthermia limited the application in tumor treatment. Therefore, it is necessary to combine the mild temperature hyperthermia with another treatment model to enhance the therapeutic effect and destroy tumors effectively [13–20].

Highly reactive hydroxyl radicals can oxidize biomolecules and induce the cancer cellular necrosis and apoptosis [21–23]. Reactive hydroxyl radicals can be produced from Fenton reaction, which utilizes Fenton active agents to catalyze the conversion H_2_O_2_ [24–27]. Moreover, cancer cells could overproduce hydrogen peroxide and improve the level of hydrogen peroxide in the tumor microenvironment (TME). Hence, various of Fenton active agents containing ferrous iron [Fe(II)] or Mn iron [Mn(II)] were exploited to generate highly reactive hydroxyl radicals for treatment of tumors [28–31]. Unfortunately, the therapeutic efficacy of Fenton reaction alone is still limited due to suppressive reaction conditions in tumor, such as low concentration of Fenton active agents. Moreover, raising temperature may be conducive to Fenton reaction according to thermodynamic molecular collision theory. Hence, combination of mild temperature photothermal therapy (PTT) and chemodynamic therapy (CDT) is an option to enhance the therapeutic effect [32, 33].

Prussian blue (PB) is a type of blue dye with low cost and simple preparation, which is clinically FDA-approved drug [34, 35]. Recently, PB nanoparticles have been widely exploited for the biological and clinical application [36–38]. For example, PB nanoparticles were utilized as T_1_ and T_2_ weighted MR imaging contrast agents [39, 40]. Furthermore, due to the strong absorption in the near-infrared region (NIR), PB nanoparticles were investigated as photothermal and photoacoustics imaging agents [41–43]. Moreover, PB nanoparticles have the potential to converse H_2_O_2_ to be highly reactive hydroxyl radicals by Fenton reaction [44, 45]. However, the MRI enhancement and photothermal therapy efficiency of PB nanoparticles still needs to be improved for clinical applications. For example, the r_1_ and r_2_ of PB nanoparticles were determined to be only 0.34 and 4.88 mM^−1^ s^−1^, respectively. On the other hand, manganese has received attention due to the bioimaging and anti-cancer activities. For example, Mn^2+^ can shorten longitudinal relaxation time of water protons and exhibit T_1_W enhancement [46]. It has been reported that incorporation Mn^2+^ into PB nanoparticles could enhance the T_1_ contrast [47, 48]. Besides, manganese demonstrates catalysis in Fenton reaction by decomposing H_2_O_2_ and acquiring the hydroxyl radicals [30, 49].

**Scheme 1:**
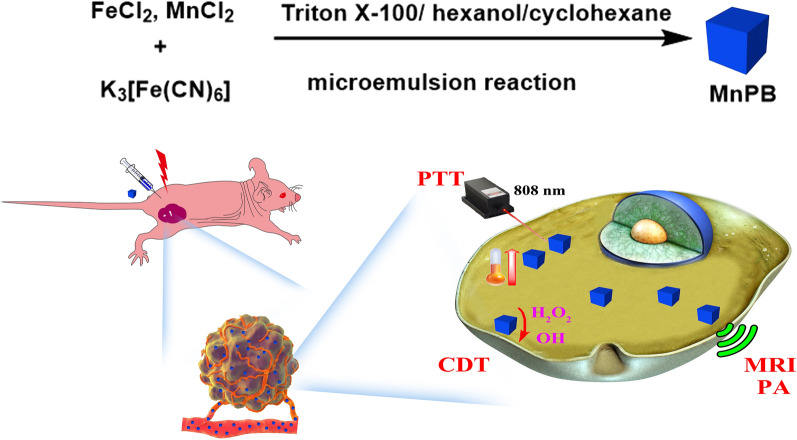
The research scheme of MnPB NPs for T_1_/T_2_ weighted MR imaging, PA imaging and CDT/PTT co-therapy in tumor

Hence, we hypothesized that incorporation Mn in PB nanoparticles can improve the multimodual imaging property and enhance the therapy efficiency of PB nanoparticles. In this contribution, we employed microemulsion method to prepare Mn doped PB nanoparticles (MnPB NPs), which are applied as T_1_ and T_2_ weighted MR imaging contrast agents (Scheme 1). Moreover, MnPB NPs demonstrated NIR absorption for mild temperature hyperthermia therapy and photoacoustic (PA) imaging. Furthermore, MnPB NPs are utilized as nanocatalysts to achieve the synergistic chemodynamic/mild temperature photothermal therapy of cancer in vitro and in vivo. Collectively, MnPB NPs exhibited excellent enhancement of multi-modal imaging in tumors, and effectively inhibited the tumor growth by combination therapy under mild conditions.

## Materials and methods

### Materials

All reagents and solvents were commercially available and used directly without further purification unless specified. CCK-8 kits were purchased from Dojindo Chemical Technology (Shanghai) Co., Ltd. (China). Calcein AM/PI Detection Kits were purchased from NanJingKeyGen Biotech Co., Ltd. (China). Annexin V-FITC/PI apoptosis kits were purchased from MultiSciences (Lianke) Biotech Co., Ltd. (China).

### Instruments

Particle size was detected by ZetasizerNano ZS (Malvern Panalytical, UK). Transmission electron microscopy (TEM) was obtained from PHILIPS TECNAI 10. UV–vis absorption spectra were measured via the Thermofisher Evolution 300 spectropolarimeter. Photoacoustic images were captured with an MSOT system (iThera Medical, Germany). Infrared thermography was captured by TiS40 infrared camera (Fluke, USA). MRI images were carried out with PharmaScan 7.0T/16 scanner (Bruker, Germany). The concentration of Mn^2+^ was measured by inductively coupled plasma-optical emission spectroscopy (ICP-OES, Thermo Scientific).

### Preparation of MnPB NPs via microemulsion method

In a typical synthesis, Triton X-100 (20 g), hexanol (10 g) and cyclohexane (10 g) were mixed to be oil EAX. Then, 8 g EAX and 3.33 g K_3_[Fe(CN)_6_] aqueous (0.05 M) were mixed and stirred for 10 min to obtain K_3_[Fe(CN)_6_] reverse microemulsion. At same time, 8 g EAX, 0.999 g MnCl_2_ (0.05 M) and 2.331 g FeCl_2_ aqueous (0.05 M) were mixed and stirred for 10 min to obtain Mn/Fe reverse microemulsion. Mn/Fe reverse microemulsion was drop added under vigorous stirring into K_3_[Fe(CN)_6_] reverse microemulsion. After 30 min, the blue nanoparticles were finally collected by centrifugation, and washed with DI water and ethanol for several times, which was denoted as MnPB NPs.

### NIR photothermal effects assessment

The NIR photothermal therapy effects of MnPB NPs were investigated as two schemes. First, 40 μg/mL MnPB NPs solution was placed in tube and irradiated under 808 nm laser with different powers (0.5, 1.0, 1.5 and 2.0 W/cm^3^). Second, MnPB NPs solutions with various concentration (0, 10, 20, 30 and 40 μg/mL) were placed in tubes and irradiated under 808 nm laser with the power of 2.0 W/cm^3^. The temperature of samples was measured via an infrared thermal imager.

### Photothermal conversion efficiency measurement

The MnPB NPs solution (20 μg/mL) was exposed to 808 nm laser (1.5 W/cm^2^) for 10 min and stop irradiated for 10 min, four heating and cooling cycles were monitored continuously, total time is 80 min.

The photothermal conversion efficiency of MnPB NPs was calculated from the first heating and cooling curve in four cycles by Roper equation.

### Fenton reaction of MnPb NPs in tube

The mixture of 0.5 mM 3,3′,5,5′-tetramethylbenzidine (TMB) and 1 mM H_2_O_2_ was added with MnPB NPs at concentration of 30, 60, and 120 μg/mL. After 30 min, The UV–vis absorption was measured. Similarly, 0.5 mM TMB, 1 mM H_2_O_2_, and 120 μg/mL MnPB NPs were mixed under different pH and measured the UV–vis absorption. After Fenton reaction, the mixture was centrifuged for the supernatant, and detected the concentration of Mn^2+^ by ICP-OES to calculate the release rate.

The mixture of 0.1 mM Methylene Blue (MB) and 1 mM H_2_O_2_ was also given with MnPB NPs at concentration of 120 μg/mL. After 30 min, the UV–vis absorption of the mixture was measured.

### Cell culture and cell viability assessment.

Skov-3, HK2 and NIH-3T3 cells were purchased from the Shanghai Cell Bank of Type Culture Collection at the Chinese Academy of Sciences. Skov-3 cells (10^4^ cells/well) were seeded in 96-well flat bottom plates and maintained in 1640 complete medium, and cultured overnight at 37 °C and 5% carbon dioxide. HK2 and NIH-3T3 were cultured in DMEM complete medium, and their culture environment were the same as Skov-3. Cell viability was evaluated by CCK-8 kits or Calcein AM/PI Detection kits.

### Animal model

All animal experiments were conducted under the guidance of the protocols approved by the local Ethical Committee in compliance with the Chinese law on experimental animals. 5 weeks old female nude mice were provided from the Animal Center of Southern Medical University (n  = 46). The mouse bearing Skov-3 tumor was established by subcutaneously injecting Skov-3 cells (1 × 10^7^) into the right hind legs of mouse.

### In vitro and in vivo T_1_ and T_2_ weighted MRI of MnPB NPs

MnPB NPs samples were prepared at the concentration of 0, 0.031, 0.063, 0.125, 0.250, and 0.500 mg/mL in 1 mL injectors and be bundling orderly for MRI.

Skov-3 cells (2 × 10^6^) were seeded in a 75 cm^2^ culture bottle. When cells reached exponential growth, the culture medium was discarded, and MnPB NPs (200 μg/mL) in culture medium was added into culture bottle and incubated for 3 h. The blank control group was replaced with new fresh medium as the previous experiments. The cells were washed entirely with PBS buffer, digested with trypsin and centrifuged, then re-suspended with 0.5% agarose solution. After cooling, the MRI images were monitored by the Bruker Biospec 7.0T MR scanner.

Skov-3 tumor-bearing mouse were anesthetized by 2% isoflurane in oxygen and placed in prone position (n  = 3). After 0, 12 and 24 h of intravenous injection MnPB NPs (100 μL, 10 mg/kg), the Bruker Biospec 7.0T MR scanner was used to capture the T_1_ and T_2_ images of tumor area.

The T_1_ value were determined using inversion recovery (IR) sequence with Rapid Acquisition with Relaxation Enhancement (RARE) readout by Bruker Biospec 7.0T MR scanner. The parameters were as follows: TR/TE  = 10,000/30 ms, TI = 10, 20, 40, 80, 120, 200, 500, 1000, 1600, 2000, 2500, 3000 and 4000 ms, a field of view (FOV) of 30 × 30 mm^2^, matrix size  = 256 × 256, RARE factor  = 8. The longitudinal relaxation rate (r_1_) was calculated via curve fitting of R_1_ (s^−1^) vs the Fe and Mn concentration (mM). The same T_1_ mapping sequence and parameters were used for MnPB NPs in phantom, in cells and in tumors.

The T_2_ value were determined using multi slices and multi echo (MSME) sequence. The parameters were as follows: TR/TE = 2500/30 ms, TE  = 90, 210, 330, 450, 570, 690, 810, 930, 1050, 1170, 1290, 1410 and 1530 ms, a FOV of 30 × 30 mm^2^, matrix size  = 256 × 256, RARE factor  = 8. The transverse relaxation rate (r_2_) was calculated via curve fitting of R_2_ (s^−1^) vs the Fe and Mn concentration (mM). The same T_2_ mapping sequence and parameters were used for MnPB NPs in phantom, in cells and in tumors.

### In vitro and in vivo PA imaging of MnPB NPs

MnPB NPs samples were prepared at the concentration of 0, 10, 30, 50, 70, 90 μg/mL, respectively. PA imaging was performed on a multispectral optoacoustic tomography (MSOT) in Vision 256-TF small animal scanner (iThera Medical GmbH, Munich, Germany).

Skov-3 cells (2 × 10^6^) were seeded in a 75 cm^2^ culture bottle. When cells reached exponential growth, the culture medium was discarded, and MnPB NPs (100 μg/mL) in culture medium was added into culture bottle and incubated for 3 h. The cells were washed entirely with PBS buffer, digested with trypsin and centrifuged, then re-suspended with 0.5% agarose solution. After cooling, the PA image was detected by the MSOT system.

Skov-3 tumor-bearing mice were in anesthesia by 2% isoflurane in oxygen and placed in prone position (n  = 3). After 0, 6, 12 and 24 h of intravenous MnPB NPs injection (100 μL, 10 mg/kg), the MSOT system was used to captured the PA images of tumor area.

### Assessment of the chemodynamic and mild temperature photothermal therapeutics effect of MnPB NPs in vitro and in vivo

For in vitro investigation: Skov-3 cells (5 × 10^3^ cells/well) were seeded on 96-well plates and incubated overnight at 37 °C incubator. The cells were incubated with MnPB NPs for 4 h at 37 °C, and then irradiated with the 808 nm laser (1.0 W/cm^2^) for 5 min. After irradiation, the treated cells were incubated for another 3 h and rinsed with PBS for further cytotoxicity assay. The standard CCK-8 assay was employed to determine the relative viabilities of treated cells. To confirm the photothermal effect, after various treatment, Skov-3 cells were incubated with a mixture of calcein AM (calcein acetoxymethyl ester) and PI (propidium iodide) for live/dead cell double staining. After rinsed with PBS, the cell samples were observed by a fluorescence microscopy (FV1200-IX83, Olympus, Japan) and FACS Calibur flow cytometer and using 488 nm laser for Annexin V-FITC and 561 nm laser for PI excitation.

For in vivo assessment, when the tumor volume on the right side increased to about 100 mm^3^ accordingly, tumor-bearing mouse were randomly divided into six groups (n  = 30), including (1) PBS: intratumoral PBS injection (100 μL). (2) H_2_O_2_: intratumoral H_2_O_2_ injection (100 μL, 400 μM). (3) MnPB NPs: intratumoral MnPB NPs injection (100 μL, 2.0 mg/kg). (4) MnPB NPs  +  NIR: intratumoral injection MnPB NPs (100 μL, 2.0 mg/kg), 10 min later, 808 nm laser irradiation (0.8 W/cm^2^, 5 min). (5) MnPB NPs  +  H_2_O_2_: intratumoral MnPB NPs injection (100 μL), 12 h later, intratumoral H_2_O_2_ injection (100 mL, 400 μM). (6) MnPB NPs  +  H_2_O_2_ + NIR: intratumoral MnPB NPs injection (100 μL, 2.0 mg/kg), 10 min later, 808 nm laser irradiation (0.8 W/cm^2^, 5 min), and 12 h later, intratumoral H_2_O_2_ injection (100 μL, 400 μM). The tumor sizes and body weight were recorded every other day after treatments.

### Statistical analysis

All data shown in this article are from at least three independent experiments and expressed as means  ±  standard deviation (SD). Analysis of variance was carried on multiple group comparisons, and the result of *p  < 0.05, **p  < 0.01 and ***p  < 0.001 were statistically significant.

## Results and discussion

### Preparation and Property of MnPB NPs

**Fig. 1:**
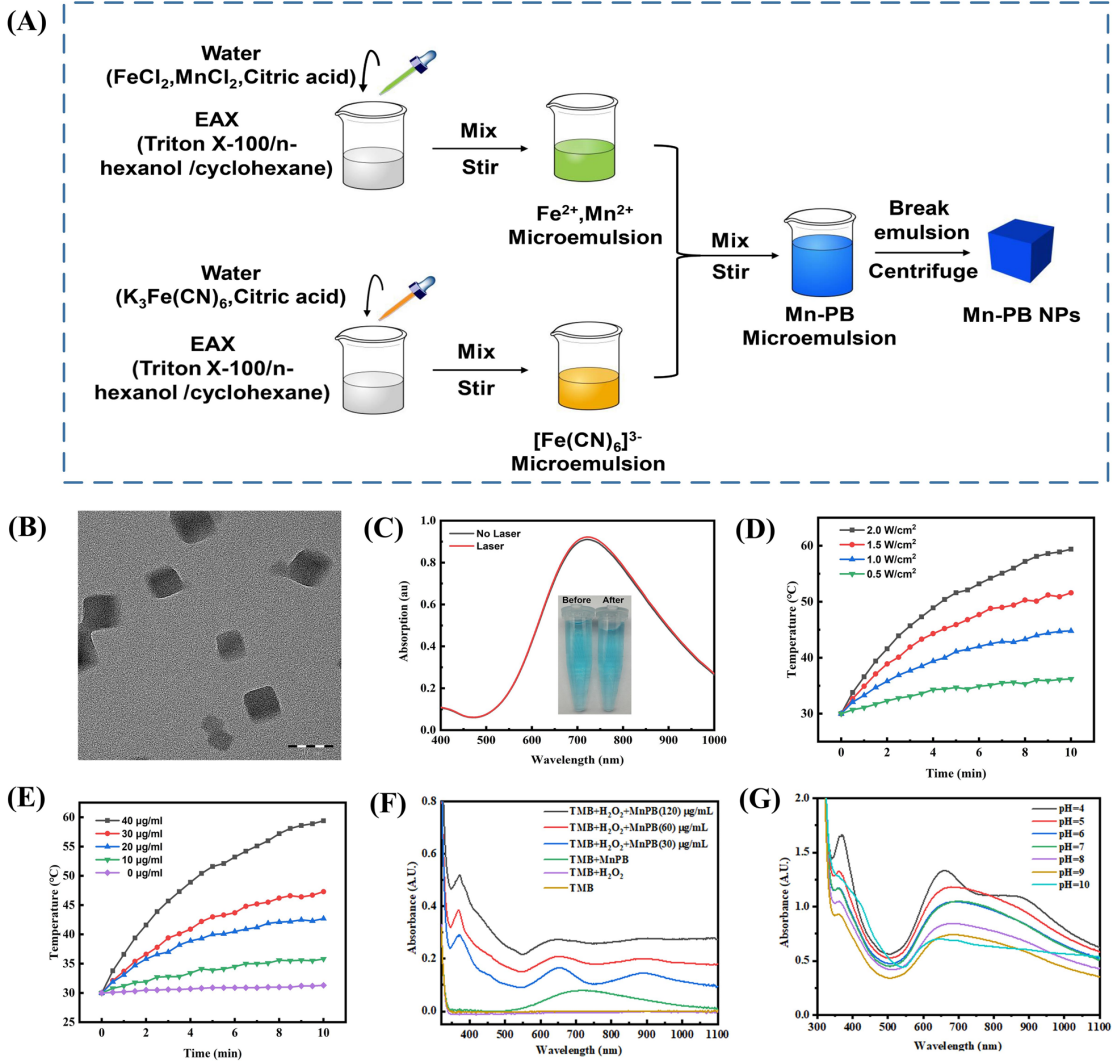
**A** The prepare routine of MnPB NPs. **B** The TEM image of MnPB NPs. **C** The UV–vis absorption of MnPB NPs before and after 808 nm laser irradiation. **D** The temperature curve of MnPB NPs under 808 nm laser irradiation with different power density. **E** The temperature curve of MnPB NPs under 808 nm laser irradiation with different MnPB NPs concentration. **F** UV–vis absorption of TMB under different condition. **G** UV–vis absorption of solution containing MnPB NPs (120 μg/mL)  + TMB (500 μM)  + H_2_O_2_ (1 mM) at different pH

Microemulsion synthesis has been proved to be a convenient method for preparation nanoparticles [50, 51]. Therefore, we prepared MnPB NPs via water-in-oil (w/o) microemulsion method as shown in Fig. 1A. TEM image of the synthesized MnPB NPs is shown in Fig. 1B. It could be found that the morphology of the nanoparticles was in cubic shape. The crystallography and phase information were acquired through X-ray diffraction (XRD), which also indicated MnPB NPs were cubic (Additional file 1: Fig. S1A). The content of Mn^2+^ and total metal ion (Mn  +  Fe) in MnPB NPs (1 mg/mL) was determined to be 0.146 mM and 1.357 mM, respectively, via ICP-OES (Additional file 1: Table S1). We then investigated the UV absorption of MnPB NPs in water (Fig. 1C). The blue MnPB NPs solution exhibited absorption peak located  ~ 710 nm, which is favorable for elimination tumor. Furthermore, the absorption spectrum kept unchanged after NIR irradiation (808 nm, 1.5 W/cm^2^) for 30 min. The dynamic light scattering (DLS) indicated MnPB NPs had an average diameter of 55 nm (Additional file 1: Fig. S1B) with zeta potential of − 26 mV and PDI of 0.21 (Additional file 1: Fig. S1C). A mass extinction coefficient of UV measured to be  ~ 9.5 cm^−1^ mg^−1^ L (Additional file 1: Fig. S1D, E). Moreover, no aggregation or precipitation in MnPB NPs solution was observed in DMEM, FBS, and H_2_O at different pH (Additional file 1: Fig. S2), verified its excellent stability.

The photothermal conversion potential of MnPB NPs was recorded under 808 nm laser irradiation. As shown in Fig. 1D, E the temperature of MnPB NPs solution increased with time and concentrations under 808 nm laser irradiation. For example, the temperature of MnPB NPs (40 μg/mL) boosted up to 63 ℃, which is favorable for ablation of tumor cells. Moreover, MnPB NPs (20 μg/mL) also demonstrated excellent photothermal performance, which reached a temperature of about 43 ℃ at such a low concentration. As shown in Additional file 1: Fig. S3A, the temperature change curve during heating and cooling of MnPB NPs solution were consistent in every cycle. Forward looking infrared radar (FLIR) thermal imaging of MnPB NPs in cuvette at different time points between 0 and 5 min under 1.5 W/cm^2^ irradiation is depicted in Additional file 1: Fig. S3B. Besides, the photothermal conversion efficiency (PCE) of MnPB NPs was calculated to be  ~ 47.38% (Additional file 1: Fig. S3C, S3D). All these results validated MnPB NPs could act as optional mediators for photothermal therapy.

### Fenton reaction of MnPb NPs in tube

3,3′,5,5′-Tetramethylbenzidine (TMB) was utilized as indicator to evaluate the catalytic activity of MnPB NPs [52]. After mixing MnPB NPs (30, 60, and 120 μg/mL) and H_2_O_2_ with TMB, the absorbance intensity at 370 and 652 nm increased (Fig. 1F). This reaction stably occurred at various pH with the 120 μg/mL MnPB NPs (Fig. 1G). After the Fenton reaction, the release rate of Mn^2+^ in supernatant was detected to be 0.88% (pH  = 7). Methylene blue (MB) also was utilized as an indicator to describe the generation of hydroxyl radicals. As shown in Additional file 1: Fig. S4, the absorption of MB in the group of MnPB NPs  +  MB  +  H_2_O_2_ was disappear, due to the oxidization of MB by hydroxyl radicals generated from Fenton reaction. Therefore, these results indicated MnPB NPs could efficiently convert H_2_O_2_ into hydroxyl radicals.

### The PTT and CDT of MnPB NPs in vitro

**Fig. 2:**
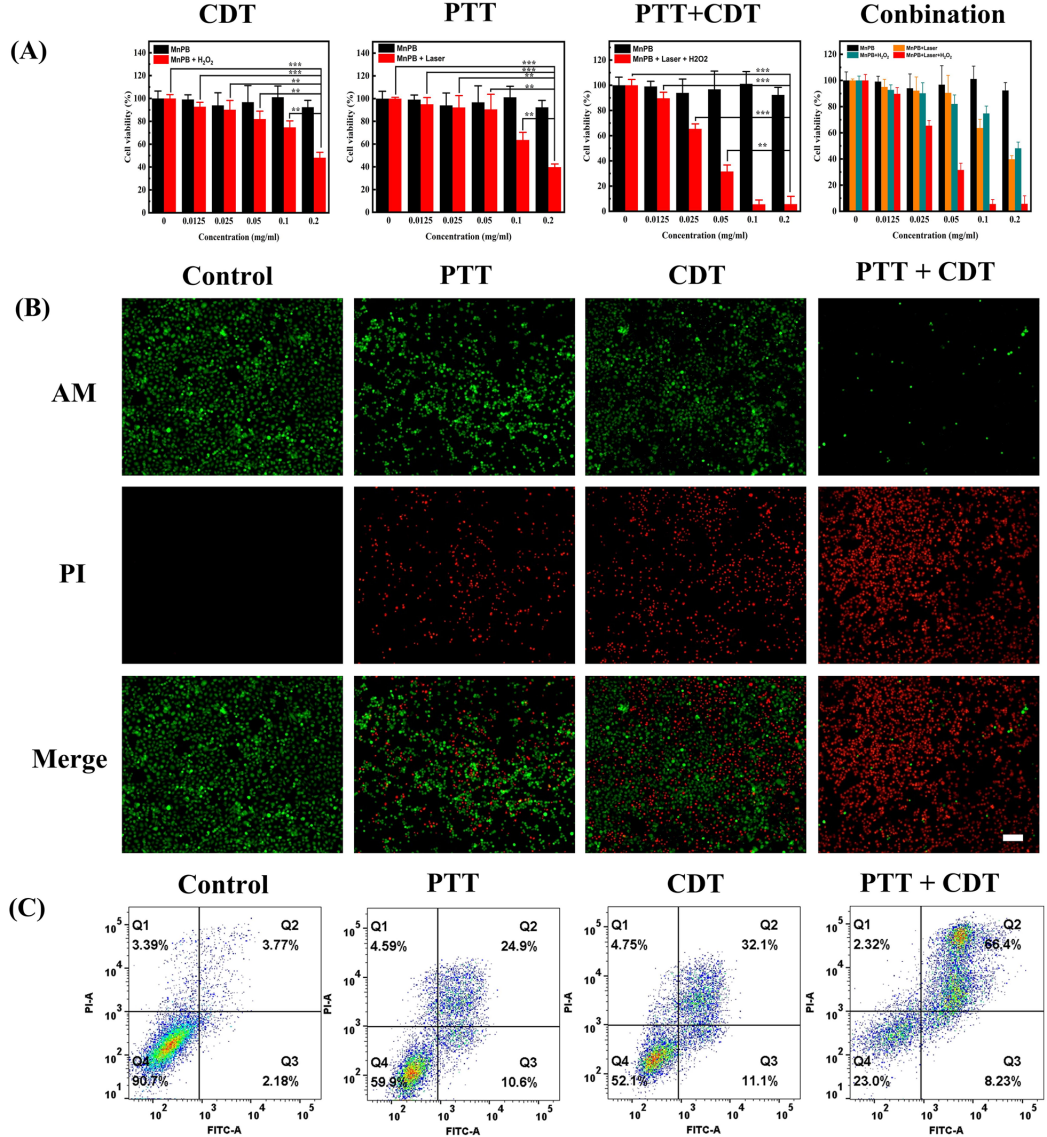
**A** The cytotoxicity of MnPB NPs under various treatment. **B** The live/dead staining images of Skov-3 cells after various treatments. Viable cells were stained green with Calcein-AM, and dead/late apoptosis cells were stained red with PI. **C** the flow cytometric analysis of Skov-3 cells after various treatments

The Cell Counting Kit-8 (CCK-8) experiment was carried out to evaluate the PTT and CDT property in vitro. As exhibited in Fig. 2A, MnPB NPs showed excellent biocompatibility and enough safety from the black bar chart. However, the cell viability diminished to  ~ 40% after treated with MnPB NPs (200 μg/mL) plus NIR irradiation (808 nm, 1.5 W/cm^2^, 10 min), which confirmed the photothermal therapy effect of MnPB NPs in cells.

Moreover, upon incubation with MnPB NPs (200 μg/mL) plus H_2_O_2_ (200 μg/mL), only  ~ 41% cells keep alive, which was ascribed to the CDT property of MnPB NPs. We then evaluated the cell viability of Skov-3 cell under treatment of incubation with 100 μg/mL MnPB NPs and 200 μg/mL H_2_O_2_, flowing with NIR irradiation (808 nm, 1.0 W/cm^2^, 10 min). To our surprise, the cell viability dropped down to 8%, which is lower compare to the 60% for PTT and 67% for CDT. The combination index (CI) between PTT and CDT was calculated to be 0.389. All these results confirmed that the strong synthetic effect of CDT and PTT of MnPB NPs.

Live/dead staining experiments were performed for further investigation the cytotoxicity and visualization of the therapeutic efficacy after various treatment (Fig. 2B). Obviously, cells treated only with MnPB NPs demonstrated bright green fluorescence with little red fluorescence. On the contrary, the cells treated by MnPB NPs (100 μg/mL) and 808 nm laser irradiation (1.0 W/cm^2^, 10 min) exhibited similar red emission intensity with the cells in MnPB NPs plus H_2_O_2_ group, which confirming the therapeutic effect of PTT or chemotherapy. Interesting, the brightest red emission with little green fluorescence were observed in the cells treated by MnPB NPs (100 μg/mL)  + 808 nm laser irradiation (1.0 W/cm^2^, 10 min)  + H_2_O_2_. All these results also proved the comminated effect of chemotherapy and PTT of MnPB NPs.

Finally, flow cytometry experiments were carried out to explore the incidence of apoptosis under different treatment. As depicted in Fig. 2C, the apoptotic rate of Skov-3 cells treated with PTT  +  CDT was determined to be 66.4%, which is superior to that in treated with PTT or CDT alone. All these data highlighted the great treatment performance of combination between PTT and CDT of MnPB NPs.

### The T_1_ and T_2_ imaging property of MnPB NPs

**Fig. 3:**
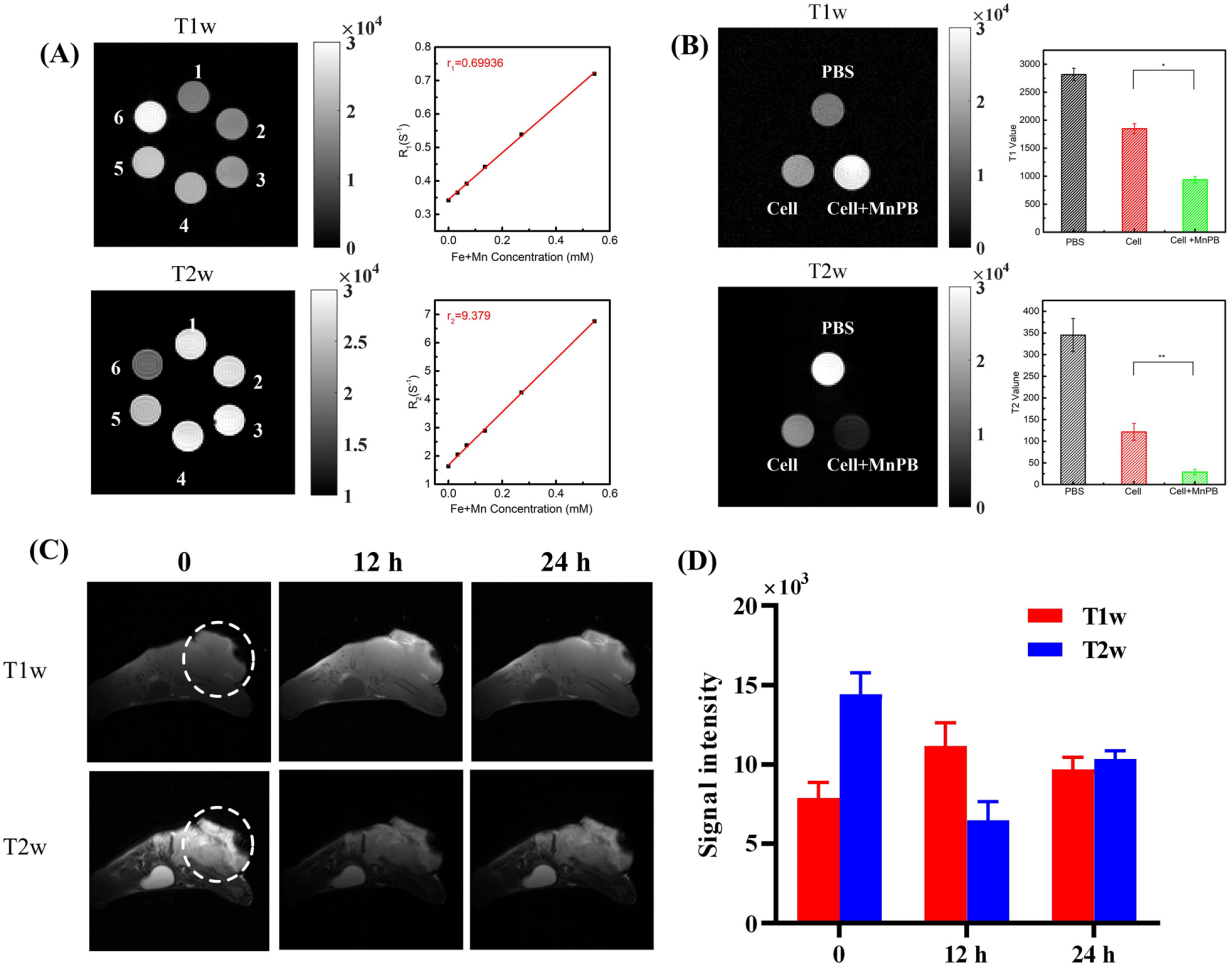
**A** T_1_ and T_2_ MR imaging of MnPB NPs. The numbers 1–6 mean the MnPB NPs concentrations arranged from 0 to 400 μg/mL. **B** T_1_ and T_2_ MR imaging of MnPB NPs in vitro. **C** T_1_ and T_2_ MR imaging of MnPB NPs in vivo (n  = 3). **D** T_1_ and T_2_-weighted MR signal intensities in the tumor before injection, 12, and 24 h post intravenously injection of MnPB NPs

The T_1_ and T_2_ imaging of MnPB NPs were then carried out. The T_1_ and T_2_ weighted MRI for MnPB NPs phantoms with concentration of 0–10 mg/mL were exhibited in Fig. 3. The T_1_W images of MnPB NPs became brighter as the concentration increases. The longitudinal and transversal relaxation ratio is linearly fitted vs the molar concentration of total metal ions and the r_1_ value of MnPB NPs was calculated to be 0.6693 mM^−1^·s^−1^, which is higher than r_1_  = 0.34 mM^−1^ s^−1^ of pure PB NPs. As demonstrated in Fig. 3A, the darkening effects for the T_2_ weighted MR images were clearly observed. The r_2_ value is measured to be 9.379 mM^−1^ s^−1^, which is larger than r_2_ (4.88 mM^−1^ s^−1^) of pure PB NPs. All these results verified the super MRI performance of MnPB NPs.

T_1_ and T_2_ weighted MR imaging of cells incubated with MnPB NPs were carried out to investigate the MR enhancement performance of MnPB NPs in vitro. Figure 3B depicted the brighter T_1_W imaging of skov-3 cells incubated with MnPB NPs with lower T_1_ value compare with that of cells in PBS group. Moreover, the darker T_2_W imaging with lower T_2_ value of cells treated with MnPB NPs was also observed. All these results indicated the excellent T_1_ and T_2_ MR enhancement of MnPB NPs in vitro.

Encouraged by the great MRI performance of MnPB NPs in vitro, MnPB NPs were intravenously injected into the Skov-3 tumor-bearing mouse. Next, in vivo T_1_ and T_2_ MR imaging were performed to verify the MR imaging property of MnPB NPs (Fig. 3C). Noticeable bright effect of T_1_W images at the tumor site was observed after injection 12 h and the quantitative MR imaging results verified that T_1_W signals obviously enhance in the tumor region (Fig. 3D). Meanwhile, the T_2_W images indicate a change of gradual dimming in the tumor after injection 12 h. To our surprise, the MRI signal in the tumor site was still noticeable even after 24 h. All the results support that MnPB NPs are feasible MRI contrast agents in living systems.

### In vitro and in vivo PA imaging

**Fig. 4:**
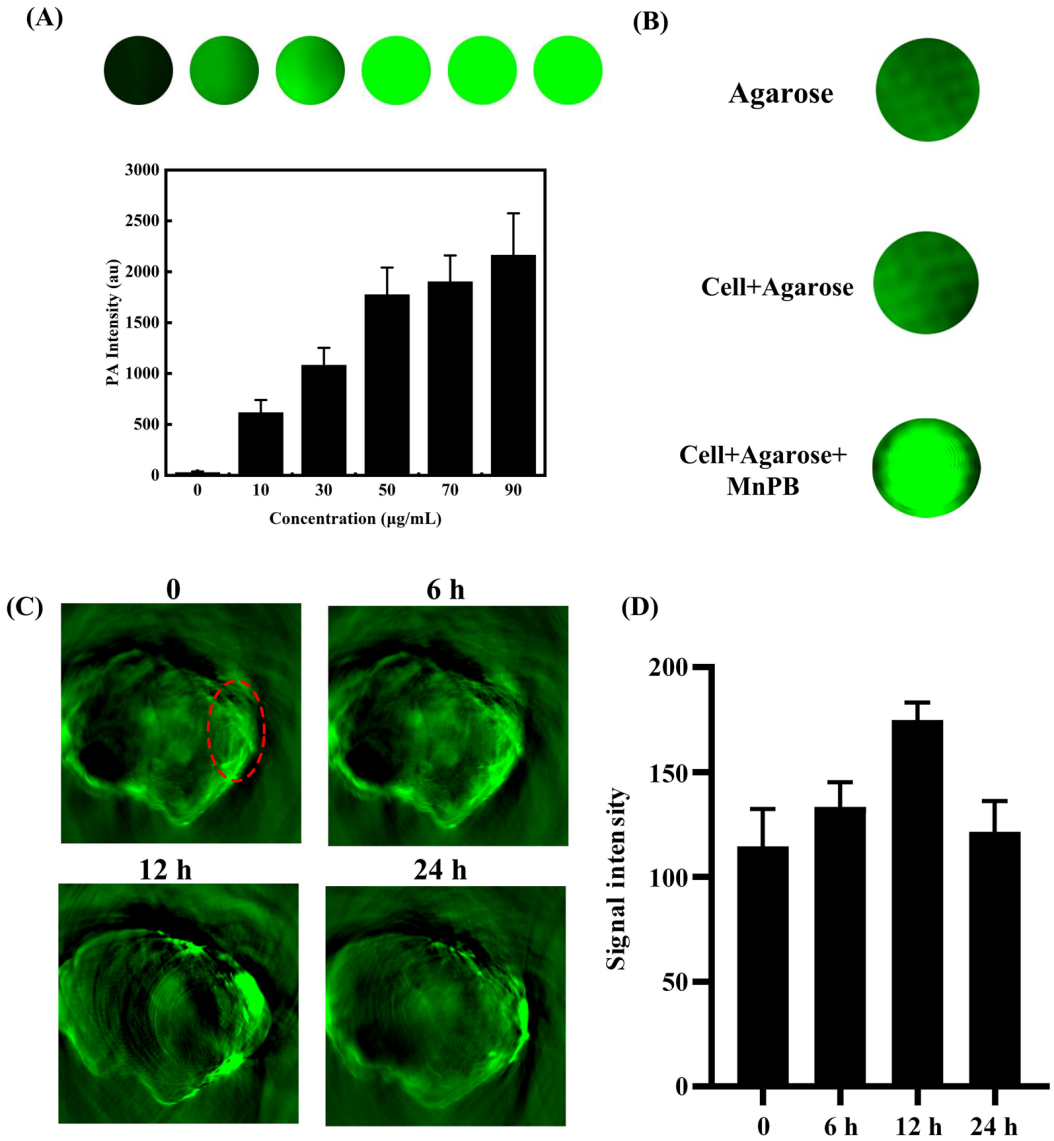
**A** Photoascoutic imaging of MnPB NPs in tube. **B** Photoascoutic imaging of MnPB NPs in vitro. **C** Photoascoutic imaging of MnPB NPs in vivo (n  = 3). **D** PA signal intensities in the tumor before injection, 6, 12, and 24 h post intravenously injection of MnPB NPs

Due to the strong absorption in NIR area, we wondered the performance of MnPB NPs as PA imaging agents. Therefore, PA images of phantoms with various concentrations were acquired and corresponding signal intensity were measured. As shown in Fig. 4A, MnPB NPs exhibited noticeable PA signal enhancement and the PA intensity increased with the increase of concentration, which proved that MnPB NPs are optional candidates for photoacoustic imaging.

Inspired by the strong photoacoustic signal in tube, photoacoustic imaging properties of MnPB NPs were further investigated in vitro (Fig. 4B). Skov-3 cells cultured with MnPB NPs (100 µg/mL) exhibited conspicuous PA signal, and confirmed MnPB NPs can be endocytosed by skov-3 cells for intracellular photoacoustic imaging. We also studied photoacoustic imaging performance of MnPB NPs in vivo. As shown in Fig. 4C, before the injection (0 min), a weak PA signal was observed due to oxyhemoglobin and deoxyhemoglobin. After intravenous injection of MnPB NPs, a strong photoacoustic signal in tumor region was detected(Fig. 4D). The PA intensity reached its highest value within 12 h. Then, with the prolongation of time, the PA signal in tumor gradually weakened, but in the tumor site, there was still a noticeable PA signal after 24 h. These results reasonably indicate that MnPB NPs can be potential photoacoustic imaging agents in vivo.

### The PTT and CDT of MnPB NPs in vivo

**Fig. 5:**
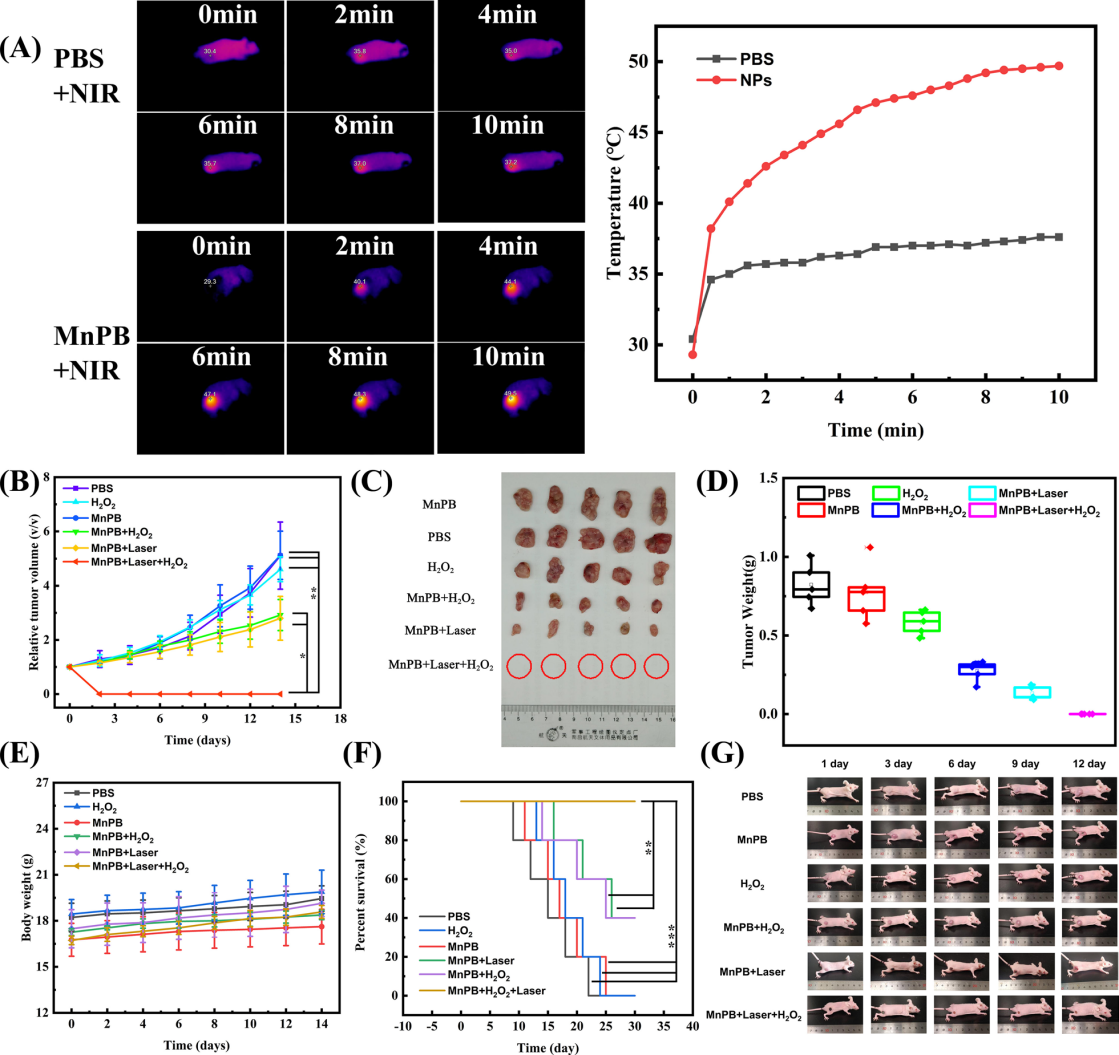
**A** The temperature of tumor tissues after 808 nm laser irradiation. **B** The tumor volume growth curves with various treatments (n  = 30). **C** The photo of dissected tumors with various treatments. **D** The weight of dissected tumors with various treatments. **E** The change curves of body weight with the different groups. **F** The percent survival of mice with various treatments during the whole experiment **G** the photos of skov-3 tumor-bearing mice after different treatment

A Skov-3 mice tumor model was established to investigate the effect of combination therapy. We first monitored the temperature changes of tumors during continuous irradiation with 808 nm laser (0.8 W/cm^2^) after injection of MnPB NPs (Fig. 5A). For example, after 10 min of irradiation, the temperature of MnPB NPs treated tumor increase from 29.3 to 49.5 °C, which is enough for ablation tumor. On the contrary, no significant temperature change of PBS treated tumor was observed under the same 808 nm laser irradiation.

Once the primary tumor size reached around 100 mm^3^, Skov-3 tumor-bearing mice were randomly divided into six groups: (i) intratumorally injection with PBS, (ii) intratumorally injection with H_2_O_2_, (iii) intratumorally injection with MnPB NPs, (iv) mild temperature PTT (intratumorally injection with MnPB NPs  +  808 nm laser irradiation), (v) CDT (intratumorally injection with MnPB NPs  +  H_2_O_2_), (vi) mild temperature PTT and CDT (intratumorally injection with MnPB NPs  + 808 nm laser irradiation  +  H_2_O_2_). The 808 nm laser irradiation was used with a power density of 0.8 W/cm^2^.

Obviously, treatment with PBS, MnPB NPs, and H_2_O_2_ alone exhibited little inhibition for the tumors. On the contrary, the tumors in the group of mild temperature PTT grow slowly, attributing to photothermal therapy (Fig. 5B). Moreover, CDT group also induced antitumor effects due to Fenton chemotherapy, which was similar to that in the group of mild temperature PTT. To our surprise, the tumors in the combination groups of mild temperature PTT  +  CDT were completely ablated, revealed that combination of these two methods achieved great therapy effect.

Finally, the tumors in each group were harvested for photographing and weighing after treatment (Fig. 5C, D). The tumor weights of the mice treated with different treatments and the photos of the tumors further confirmed the efficient of chemodynamic/mild temperature photothermal co-therapy to inhibit the tumor growth (Fig. 5E, G). Notably, the mice in the group treated with combination therapy (CDT  +  mild temperature PTT) survived more than 30 days, which was remarkably longer than other groups (Fig. 5F). These results indicated the advanced anti-tumor therapeutic efficacy of MnPB NPs through a combination of CDT and mild temperature PTT.

### The biosecurity of MnPB NPs

**Fig. 6:**
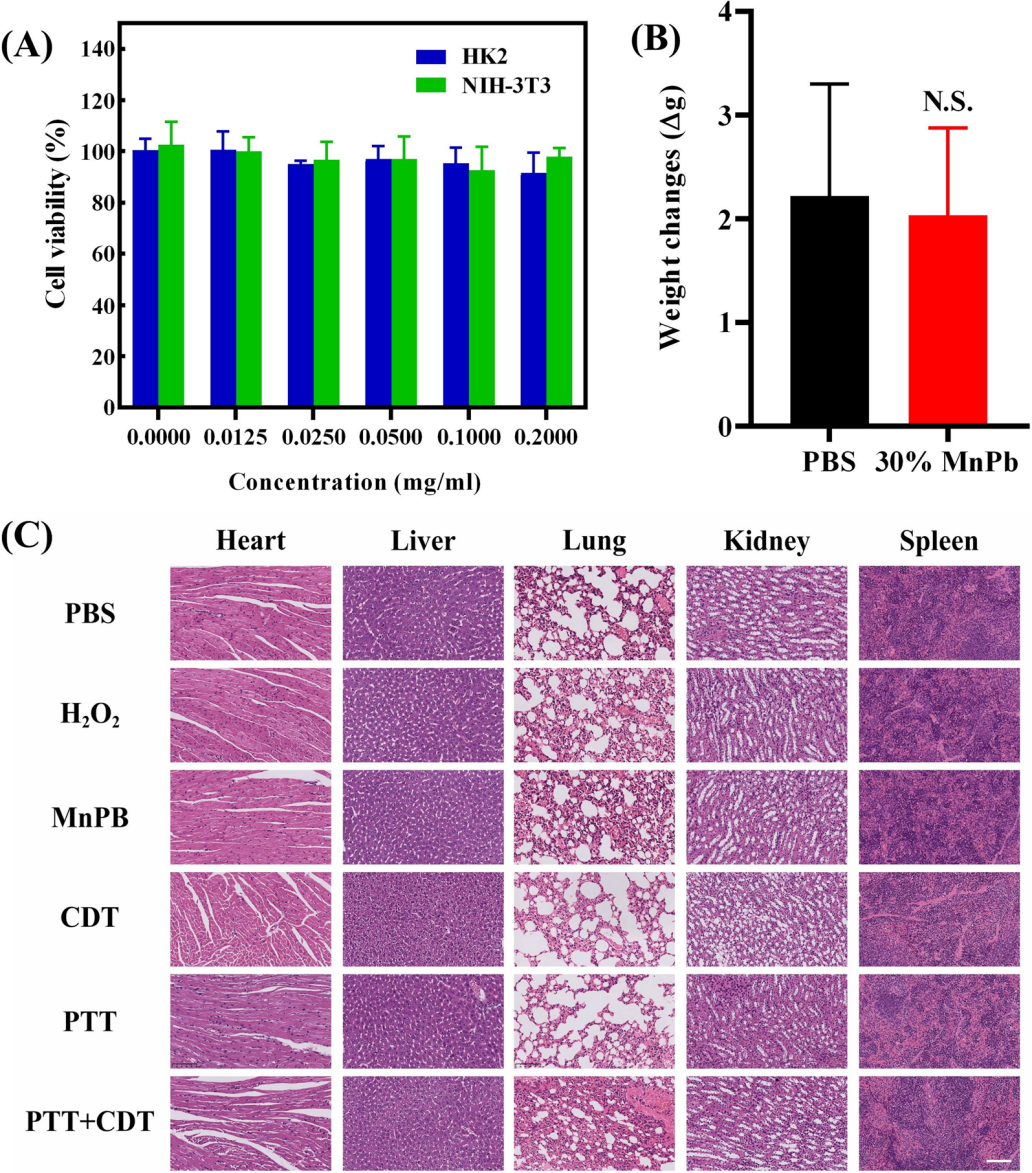
**A** The cell viability of HK2 and NIH-3T3 treated with MnPB NPs. **B** The body weight changes of mouse after been given the MnPB NPs 14 days (n  = 6). **C** The H&E staining of the major organs in different groups of MnPB NPs treated after 14 days

To investigate the biosecurity of MnPB NPs, we first explored the cytoxicity of MnPB NPs to normal cells. HK2 and NIH-33 were chosen as normal cells. As shown in Fig. 6A, there are no significant change of cell viability even the concentration of MnPB NPs reach 0.2 mg/mL, which confirmed the excellent biocompatibility to normal cells. We also recorded the body weight of normal mice after intravenous injection of MnPB NPs or PBS. As demonstrated in Fig. 6B, the body weight changes between these two groups exhibited no significant difference. At the same time, the H&E staining photos of different organ in different groups showed no obvious inflammatory reaction and changes of morphology, especially in MnPB NPs, CDT, and PTT groups. All these results indicated that the great biosecurity of MnPB NPs and the treatment methods.

## Conclusion

In this contribution, innovative Mn-doped Prussian blue nanoparticles (MnPB NPs) were prepared via a microemulsion method. MnPB NPs exhibited excellent T_1_ and T_2_ weighted MRI enhancement in vitro and in vivo, also showed robust NIR absorbance, which bring high antitumor efficacy of photothermal therapy (PTT) and provide great photoacoustics imaging enhancement. Moreover, MnPB NPs demonstrated excellent CDT efficacy. In vivo experiment verified the favorable trimodal imaging and synergistic therapy. Overall, this Mn doped Prussian blue nanoplatform can be used for multi-modal imaging and co-therapy of CDT and mild temperature PTT, which provides a reliable tool for tumor treatment.

## Supplementary Information

**Additional file 1: Table S1.** The doping ratios of Mn in final MnPB NPs products. **Figure S1.** **A** The XRD of MnPB NPs. **B** The DLS of MnPB NPs. **C** The zeta potential of MnPB NPs. **D** The UV-vis absorption of MnPB NPs with different concentration. **E** The mass extinction coefficient of MnPB NPs. **Figure S2.** **A** The stable average diameter and PDI of Mn PB NPs changes with time. **B** The size of Mn PB NPs in water with different pH. **C** The stability of Mn PB NPs in H_2_O, FBS, and DMEM. **Figure S3.** **A** The heating and cool cycle of MnPB NPs. **B** The IR imaging of MnPB NPs under 808 nm laser irradiation. **C** Time constant for heat transfer of MnPB NPs. **D** Time constant for heat transfer of water. **Figure S4.** UV-vis absorption of different experimental groups to test the Fenton reaction.

## Data Availability

All data generated or analyzed during this study are included in this published article.
