# Supplementary material for: Mn doped Prussian blue nanoparticles for T1/T2 MR imaging, PA imaging and Fenton reaction enhanced mild temperature photothermal therapy of tumor
Source: J Nanobiotechnology. 2022 Jan 4;20:18. doi: 10.1186/s12951-021-01235-2 (PMC8725273; doi:10.1186/s12951-021-01235-2)
Supplement: Supplementary file 1 — Additional file 1: Table S1. The doping ratios of Mn in final MnPB NPs products. Figure S1. A The XRD of MnPB NPs. B The DLS of MnPB NPs. C The zeta potential of MnPB NPs. D The UV-vis absorption of MnPB NPs with different concentration. E The mass extinction coefficient of MnPB NPs. Figure S2. A The stable average diameter and PDI of Mn PB NPs changes with time. B The size of Mn PB NPs in water with different pH. C The stability of Mn PB NPs in H2O, FBS, and DMEM. Figure S3. A The heating and cool cycle of MnPB NPs. B The IR imaging of MnPB NPs under 808 nm laser irradiation. C Time constant for heat transfer of MnPB NPs. D Time constant for heat transfer of water. Figure S4. UV-vis absorption of different experimental groups to test the Fenton reaction. [file 12951_2021_1235_MOESM1_ESM.docx]

Supporting information

**Mn dopped Prussian Blue nanoparticles for T_1_/T_2_ MR imaging, PA imaging and Fenton reaction enhanced mild temperature photothermal therapy of tumor**

Quan Tao,Genghan He, Sheng Ye, Di Zhang, Zhide Zhang, Li Qi, Ruiyuan Liu

**Content**

Table S1. The doping ratios of Mn in final MnPB NPs products.

Figure S1. (A) The XRD of MnPB NPs. (B) The DLS of MnPB NPs. (C) The zeta potential of MnPB NPs. (D) The UV-vis absorption of MnPB NPs with different concentration. (E) The mass extinction coefficient of MnPB NPs

Figure S2. (A) The stable average diameter and PDI of Mn PB NPs changes with time. (B) The size of Mn PB NPs in water with different pH. (C) The stability of Mn PB NPs in H2O, FBS, and DMEM.

Figure S3. (A) The heating and cool cycle of MnPB NPs. (B) The IR imaging of MnPB NPs under 808 nm laser irradiation. (C) Time constant for heat transfer of MnPB NPs. (D) Time constant for heat transfer of water.

Figure S4. UV-vis absorption of different experimental groups to test the Fenton reaction.

|  | **FeCl_2_**  **(M)** | **MnCl_2_**  **(M)** | **K_3_[Fe(CN)_6_]**  **(M)** | **Mn/(Mn+Fe)** | **Mn/MnPb NPs** |
| --- | --- | --- | --- | --- | --- |
| PB | 0.05 | 0 | 0.05 | 0 | 0 |
| MnPB NPs | 0.05 | 0.05 | 0.05 | 10.73% | 5.37% |

Table S1. The doping ratios of Mn in final MnPB NPs products.


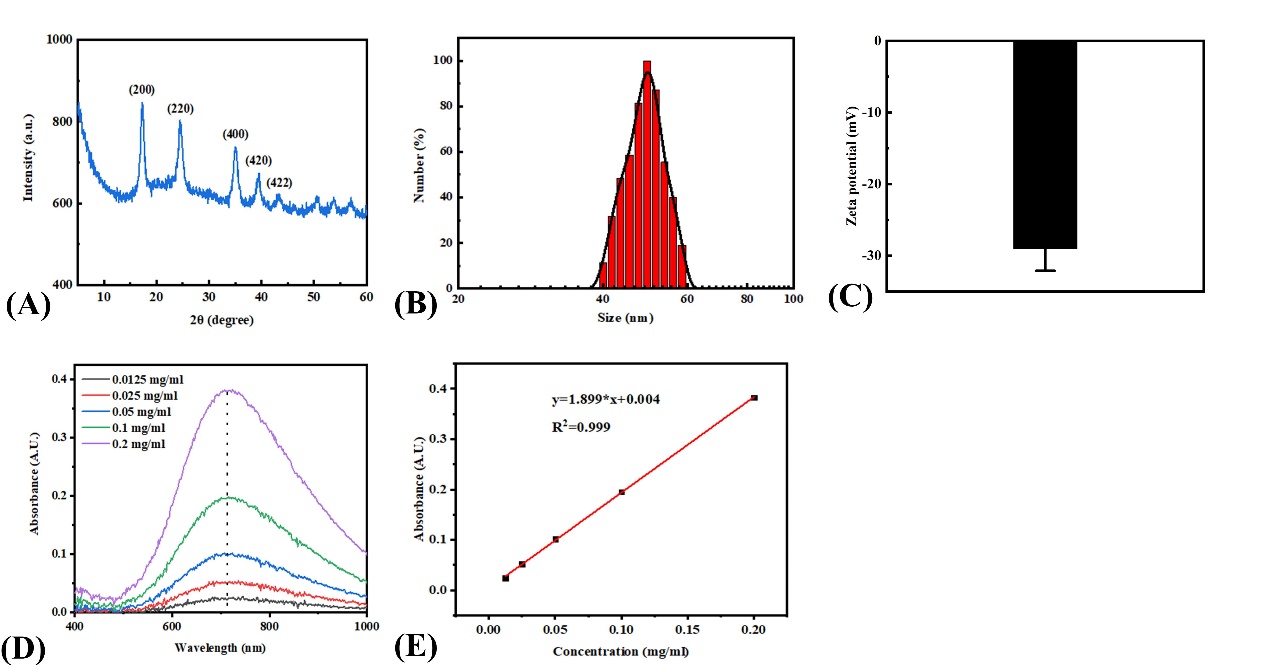


Figure S1 (A) The XRD of MnPB NPs. (B) The DLS of MnPB NPs. (C) The zeta potential of MnPB NPs. (D) The UV-vis absorption of MnPB NPs with different concentration. (E) The mass extinction coefficient of MnPB NPs


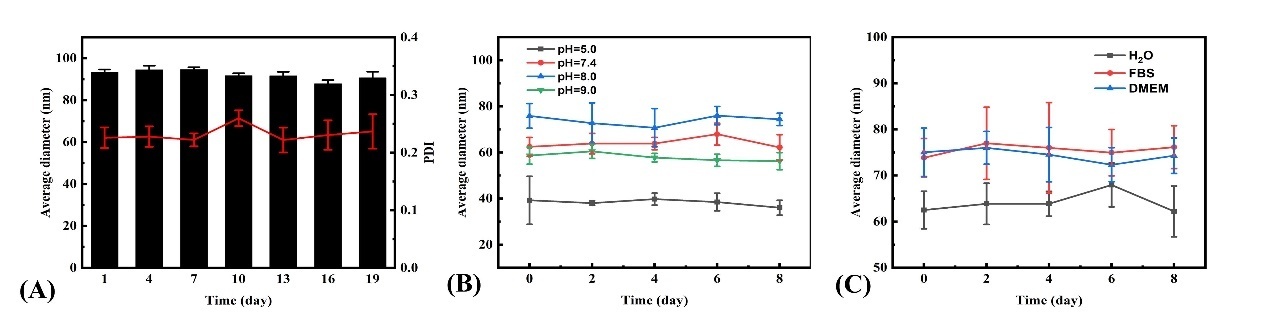


Figure S2 (A) The stable average diameter and PDI of Mn PB NPs changes with time. (B) The size of Mn PB NPs in water with different pH. (C) The stability of Mn PB NPs in H2O, FBS, and DMEM.


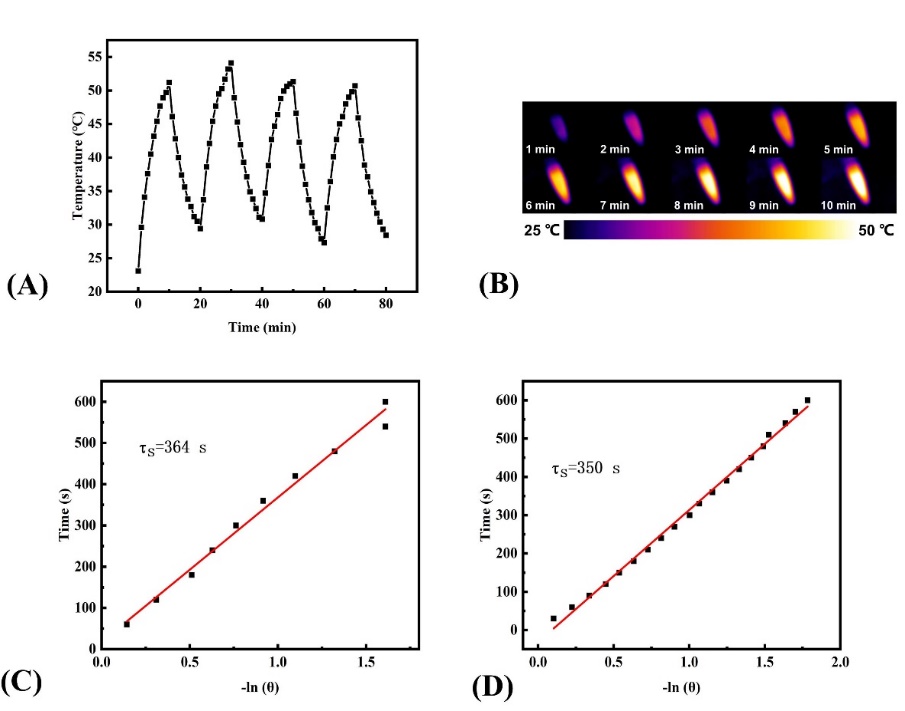


Figure S3. (A) The heating and cool cycle of MnPB NPs. (B) The IR imaging of MnPB NPs under 808 nm laser irradiation. (C) Time constant for heat transfer of MnPB NPs. (D) Time constant for heat transfer of water.


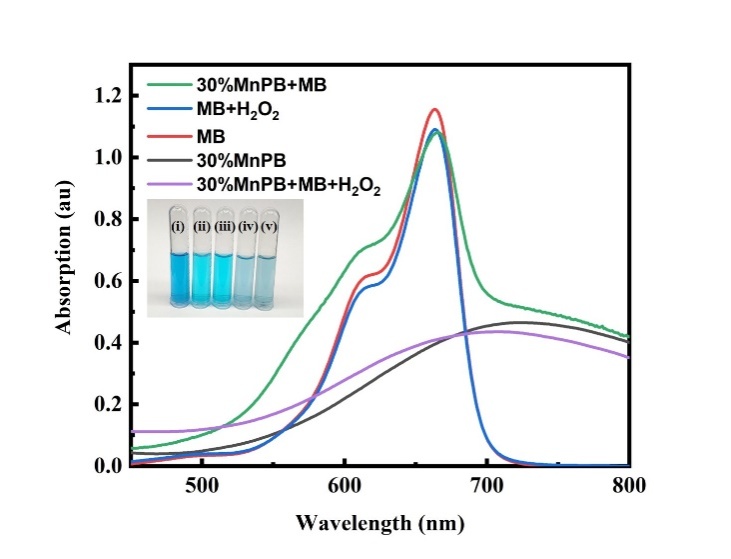


Figure S4. UV-vis absorption of different experimental groups to test the Fenton reaction.
